# An Eye-Tracking Version of the Trail-Making Test

**DOI:** 10.1371/journal.pone.0084061

**Published:** 2013-12-18

**Authors:** Stephen L. Hicks, Rakesh Sharma, Amad N. Khan, Claire M. Berna, Andrea Waldecker, Kevin Talbot, Chris Kennard, Martin R. Turner

**Affiliations:** Nuffield Department of Clinical Neurosciences, University of Oxford, Oxford, United Kingdom; University of Iowa Carver College of Medicine, United States of America

## Abstract

The neurodegenerative disorder amyotrophic lateral sclerosis may render patients unable to speak or write, so that objective assessment of cognitive impairment, which is commonly of a dysexecutive nature, is challenging. There is therefore a need to develop other methods of assessment that utilize other relatively unaffected motor systems. In this proof-of-principle study a novel eye-tracking version of the trail-making test was compared with performance on the standard written version in a group of healthy volunteers. There was good correlation for speed between both versions of Part B (R^2^=0.73), suggesting that this is a viable method to objectively assess cognitive impairment in disorders where patients are unable to speak or write.

## Introduction

The trail-making tests (TMTs) are neuropsychological assessments of visual attention, psychomotor processing speed, and task-switching. The test was developed from the Taylor Number Series Test, which required the subject to connect a randomly distributed set of numbers sequentially from 1 to 50 [1]. The most widely used version of the TMT comprises parts A and B which are both timed in seconds to completion [2]. In part A, the subject uses a pencil to connect a series of 25 encircled numbers in numerical order (1, 2, 3…). In part B, the subject connects 25 encircled numbers and letters in numerical and alphabetical order, alternating between the numbers and letters (1, A, 2, B, 3, C, 4, D…). The numbers and letters are placed in a semi-random fixed order, in such a manner as to avoid overlapping lines being drawn by the examinee. While part A is generally presumed to be a test of visual search and motor skills, part B is considered to be a test of higher level cognitive skills such as mental flexibility [3-5].

Electroencephalography has implicated frontal lobe activation during performance on the TMT [6], while a study using a verbal adaptation of the TMT [7] found that the set shifting component (1, A, 2, B, 3, C,...) activated the dorsolateral prefrontal cortex (DLFPC) and supplementary motor area, postulated to be sensitive to executive functioning, particularly cognitive flexibility. Functional magnetic resonance imaging demonstrated activation of the left DLPFC, precentral gyrus, cingulate gyrus and medial frontal gyrus during part B [8].

Amyotrophic lateral sclerosis (ALS) is a progressive neurodegenerative condition and has clinical, pathological and genetic overlap with frontotemporal dementia [9]. Cognitive involvement in ALS is characterised by consistent deficits in executive function, which carry adverse prognosis [10]. The recognition of ALS as a multi-system cerebral disorder has made neuropsychological assessment a routine part of clinical research, but its application is limited by the frequent loss of use of writing and speech functions as a result of disease progression. Motor neurons subserving oculomotor function, in contrast, are largely preserved throughout the course of ALS [11]. The wider cerebral networks involved in saccade generation, particularly in the frontal lobes, in conjunction with the increased sophistication and practicality of eye-tracking equipment, make eye movements a potentially uniquely practical method of studying extramotor cerebral pathology in ALS. While language deficits [12], particularly verbal fluency [13], appear to be the most consistently impaired neuropsychological tests in ALS patients, TMT performance is also affected [14,15], and has been specifically linked to widespread frontal lobe cerebral white matter changes [16].

Among the wide range of neuropsychological tests sensitive to ALS pathology [17], the TMT was the most obviously transferrable to the hands-free, speech-free environment. We therefore developed an oculomotor-driven version of the TMT and compared it to the traditional written version in a group of healthy volunteers.

## Methods

### Ethics statement

The study was approved by the London Riverside Research Ethics Committee (04/q/0406/60). Written informed consent was obtained from all participants, and all research was carried out in UK.

### Participants

Forty healthy volunteers aged between 22 and 52 years (mean age 28±7) with no significant past medical or psychiatric histories were recruited (21 females, 19 males). Each participant performed both written and oculomotor versions of the TMT. To minimize practice effects, half of the subjects performed the written version of the test first, followed one week later by the oculomotor version, and *vice versa* for the other half. 

### Written TMT

The written TMT was administered according to standard protocol [2]. Participants were instructed to draw a continuous line on the paper “quickly and accurately” connecting the stimuli. If a subject made a mistake during the test and did not correct it, the study investigator then immediately corrected them. The total time to connect all 25 items on both sheets (A and B) was measured, including the extra time required for the administrator to correct for errors.

### Oculomotor TMT

A horizontal flipping of the written trail was used, keeping the arrangement of items intact and rotated by 90 degrees to accommodate the landscape screen format (resolution 1280 x 1024 pixels). The stimuli (letter or number) were included into contingent squares of 150 pixels. An EyeLink-1000 infrared pupil tracker with EyeLink Experiment Builder® software was used to record fixation accuracy for all participants throughout all experimental conditions. Eye movement data were analysed off-line using Experiment Builder (SR research) and custom programs written in Matlab. Head position was stabilized using a chin rest and the distance to the screen was 57 centimeters. A nine-points calibration covering the totality of the visual screen and a drift correction was carried out before each test (A/B) to ensure an accurate eye position recording.

In order to select an item on the computer screen, participants were required to fixate on the item for a minimum of 400 milliseconds. This delay was designed to avoid unintended activation during visual scanning. When the item was fixated, the ring surrounding the number or letter turned from white to red, as a signal for the participant to move to the next item in the sequence. Only the correct item in the sequence could be selected (and turn red), which made it possible to score an attempt to select an item as an error (see Video S1). Once an item was selected, the previous item in the sequence turned from red to dark grey to mark it as completed.

As per standard procedure in the written TMT, subjects were allowed to practice the oculomotor-based tests once immediately before the testing, using Parts A & B with fewer items and a different spatial arrangement. This also allowed subjects to experience the concept of gaze contingency (the online response of the program to eye position) and the fixation duration required to select the correct item (400ms) in a sequence.

To demonstrate a difference in cognitive load between Parts A & B, the number of fixations between targets, i.e. the number of different targets subjects gazed at before arriving upon the right target, was examined.

## Results

**Figure 1:**
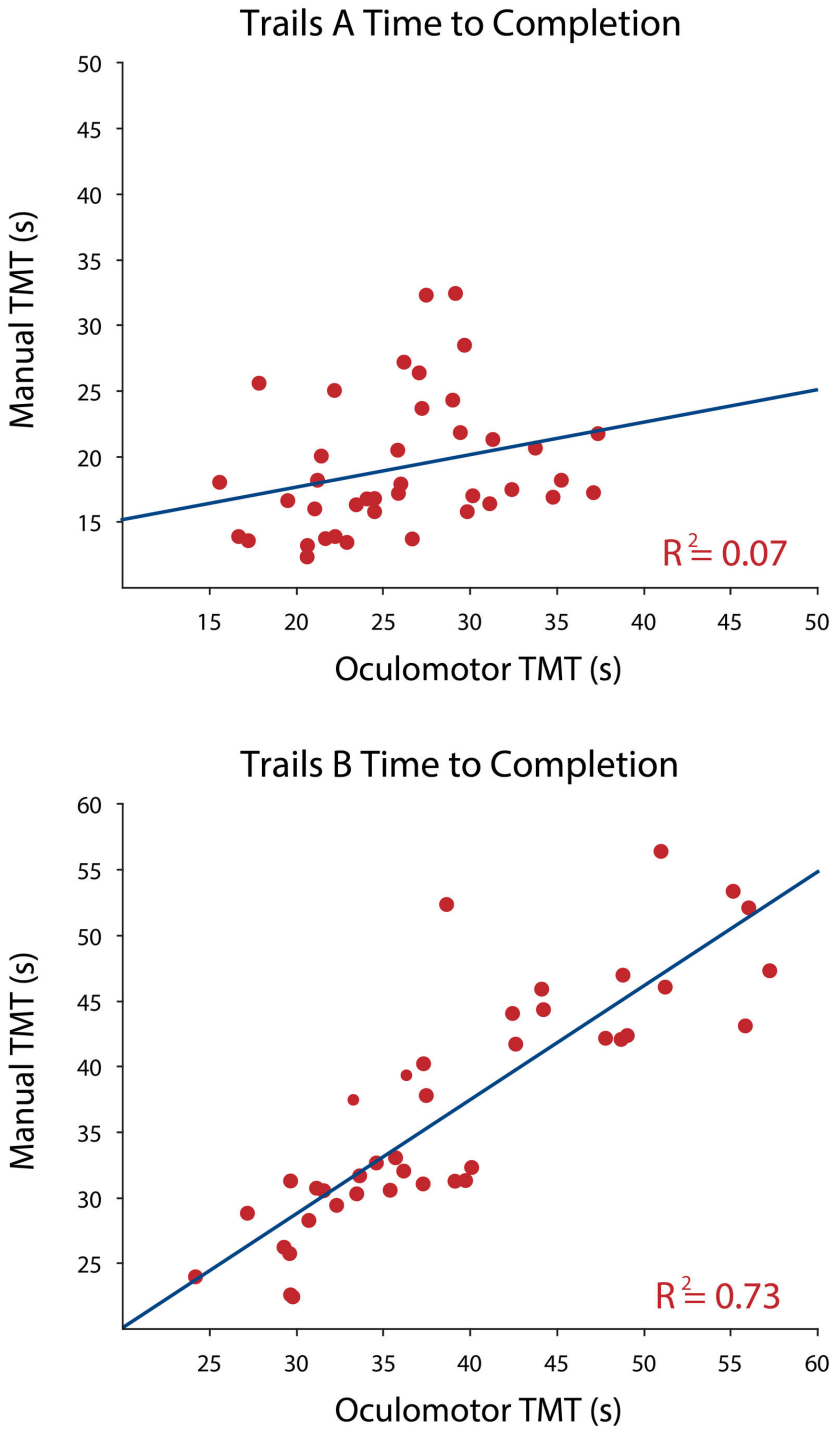
Scatterplots showing correlations between the written (y axis) and oculomotor versions of the trail-making test. There is a strong correlation for Part B (lower panel) but none for Part A (upper panel).

**Figure 2:**
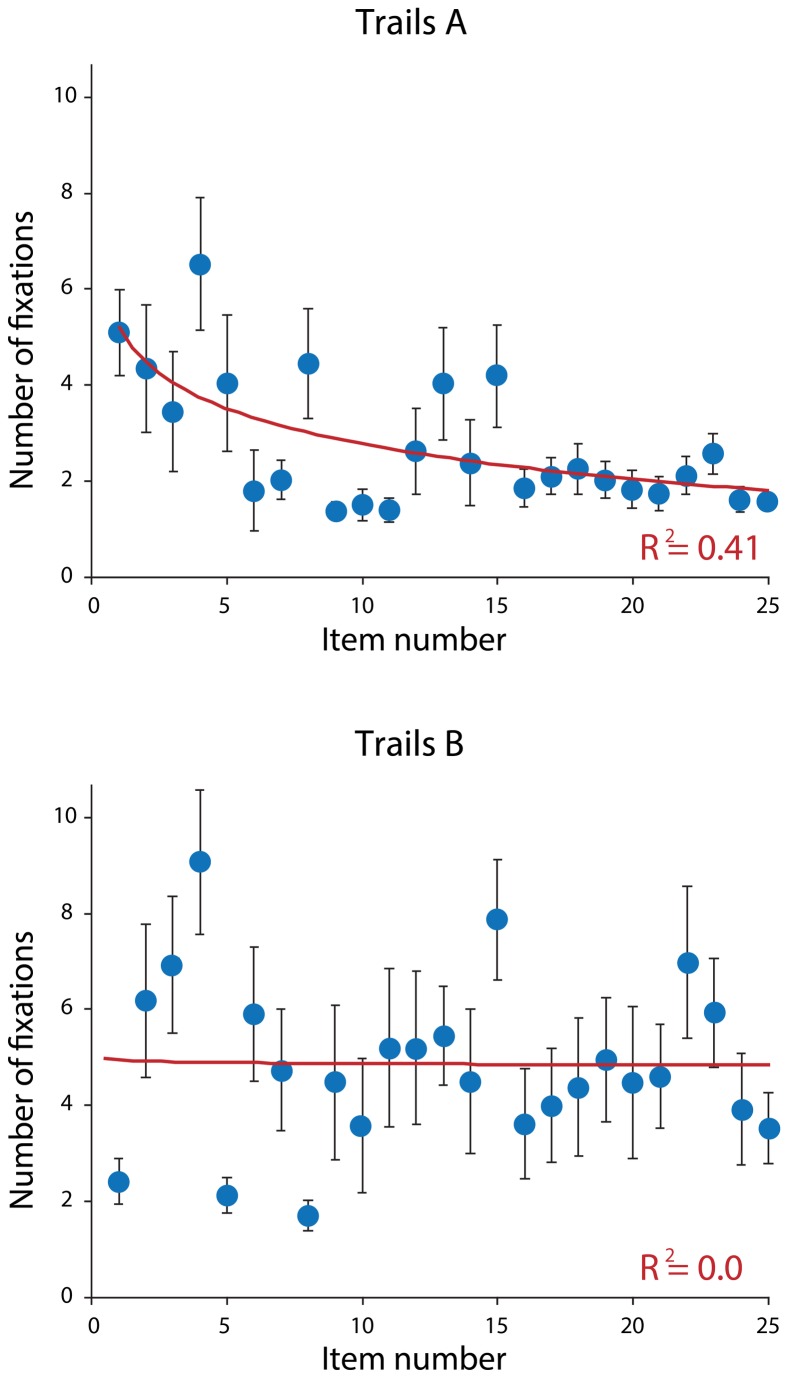
Scatterplots showing the number of fixations recorded for each target during the oculomotor trail-making tests. This decreased significantly during Part A (upper panel), but remained static during Part B (lower panel).

A linear regression analysis was performed on the data to determine whether scores on the written TMT could predict scores on the oculomotor TMT. There was good correlation between total time taken to complete the written versus oculomotor version of Part B (R^2^=0.73), though no correlation for Part A (R^2^=0.07) was revealed (Figure **1**). There was no effect for age or gender in part A (p=0.74 and p=0.23 respectively), or part B (p=0.65 and p=0.44 respectively). The number of fixations decreased progressively for Part A (R^2^=0.41), but not in Part B (Figure **2**).

## Discussion

This study demonstrated that an oculomotor-driven version of the TMT can be applied to healthy volunteers, and in which Part B performs comparably to the standard written version. Notwithstanding the need for formal reliability and validation testing, it has potential to explore dysexecutive functioning in those unable to write or speak, and broadens the potential application of eye-tracking to encompass tests of cognitive function in ALS.

The striking disparity between the correlations of Trail A and Trail B between paradigms was unexpected, although this was also observed in a comparative study of the oral and written versions of the TMT [18]. Like these authors, we attribute the lack of correlation between the written and oculomotor version of Part A to the low cognitive input required for this part of the test, so that variability due to individual preference for method (written versus eye-tracking) was more apparent. Part B has inherently high cognitive demands [5], that may mask any variance attributable to input methodology. This is supported by the progressive drop in fixations seen in Part A but not Part B of the oculomotor version. As participants progressed through the task, the number of available items decreased, reducing the number of fixations required to select the remaining targets. In Part A, this is seen as a steady reduction in oculomotor output, while in Part B the high number of fixations at the end of the trial may be the result of the increased cognitive load interfering with the participants’ search strategy. We were not able to quantify errors for the eye-tracking version of the test, though a systematic difference between the two methods seems unlikely.

Several variations of the TMT have been developed over the years. The Color Trails Test [19] was developed to accommodate testing in subjects with language difficulties, although there is some evidence that the Part B equivalent of this test is not compatible with the original TMT Part B, described as ‘culture-loaded’ because of its reliance on the Latin alphabet. It has been demonstrated that it is particularly unreliable in younger and less educated subjects [20].

An oral version of the TMT was developed and may distinguish between spatial and motor components from sequencing deficits when used in conjunction with the standard written test [21]. It has been used in conditions like ALS where there is significant limb impairment, and it would be useful to directly compare the performance of the oral version alongside the eye-tracking one. Although novel analogues of the classic TMT have been found to be effective alternatives [22], in general computerized and paper-and-pencil versions of the test have not been found to be equivalent before now [23], possibly because of the variability of the motor demand of the computer interface [24]. The gaze-contingent paradigm on which the experiment here is built minimizes the motor component. Furthermore, subjects are not expected to speak during the test either. Only eye movements are used to fixate and search targets on the screen in an intuitive way.

Portable eye-tracking devices have made a valuable contribution to the study of neurodegenerative diseases such as Huntington’s disease [25]. Provided oculomotor functions are mechanically preserved, it is potentially possible to use this paradigm to test other cognitive domains in subjects who are unable to speak or write. The increased sophistication of eye-tracking equipment, means it is possible also to delineate the nature of errors that patients make while scanning targets, such as perseverative errors (the tendency to insist on staring at a selected item reflecting a lack of flexibility), errors due to disinhibition (the inability to maintain gaze for the required 400 ms) and sequencing errors (the inability to maintain two separate sets in working memory during Part B). This might allow more subtle exploration of cognitive dysfunction across a range of neurological disorders.

## Supporting Information

Video S1 **Video showing administration of the trail-making test Part B using the eye-tracking equipment.** The subject’s head is apposed comfortably in the frame at a fixed distance from the screen. The target being fixated turns red and its border fades to grey to denote successful sequencing. As the camera pans to the left, the administrator’s screen is seen which shows the subject’s progress, followed by a screen showing the position of visual fixation at any moment as a white moving cursor.
(MOV)
